# Crosstalk of cancer stemness-neutrophils in outcome of intracranial germ cell tumors

**DOI:** 10.3389/fimmu.2026.1571513

**Published:** 2026-03-05

**Authors:** Botao Zhang, Qiang Ji, Yi Lin, Wenbin Li

**Affiliations:** Department of Neuro-oncology, Cancer Center, Beijing Tiantan Hospital, Capital Medical University, Beijing, China

**Keywords:** cancer stemness, crosstalk, intracranial germ cell tumors, neutrophils, outcome

## Abstract

**Background:**

Intracranial germ cell tumors (iGCTs) are categorized into germinomas (GEs) and non-germinomatous germ cell tumors (NGGCTs), which show divergent clinical outcomes with GEs having a significantly more favorable prognosis. This prognostic disparity suggests distinct biological characteristics, yet the association between tumor stemness and the host immune microenvironment, particularly in pre-treatment peripheral blood, remains poorly understood. Therefore, this study integrated multi-omics data, immune cell enrichment scores, and peripheral blood analyses to elucidate the relationship between cancer stemness indices and immune cells, especially neutrophils; and to evaluate its prognosis and treatment strategy in iGCTs.

**Methods:**

We included iGCT patients from Tiantan Hospital, all of whom underwent peripheral blood testing before pre-chemotherapy. In addition, gene expression microarray, 450K methylation data, and copy number variation data were obtained from the Gene Expression Omnibus (GEO) database. Bioinformatics analysis was used to assess and compare stemness, biological functions, immune microenvironment characteristics between GEs and NGGCTs. Furthermore, we specifically investigated the correlation between stemness indices and neutrophil levels in both subtypes.

**Results:**

Our analysis revealed that the stemness of GEs was significantly higher than that of NGGCTs. Functional enrichment analysis indicated that GEs are primarily involved in processes related to cell proliferation, whereas NGGCTs were mainly associated with extracellular matrix processes. Patients with GEs had better prognoses than those with NGGCTs, highlighting the crucial role of the tumor microenvironment on tumor progression. The immune landscape also differed substantially between the two subtypes, with distinct patterns of immune cell infiltration and response. Notably, the correlation between cancer stemness and neutrophil levels showed the most marked difference between GEs and NGGCTs. Our data further validated that neutrophil abundance is positively associated with increased malignancy in iGCTs, suggesting that neutrophils represent a key factor influencing iGCT outcomes.

**Conclusion:**

Our study demonstrates that the interaction between neutrophils and stemness contributes to the malignant progression of iGCTs, leading to adverse clinical outcomes. These findings not only advance our understanding of iGCT biology but also highlight potential novel therapeutic targets. Moreover, they provide a rationale for developing immunotherapy strategies aimed at modulating the neutrophil-stemness axis in iGCTs.

## 1 Background

Intracranial germ cell tumors (iGCTs) are a group of rare, heterogeneous malignant neoplasms of the central nervous system (CNS), occurring primarily in children and adolescents. In China, they represent 8.1% of primary pediatric CNS tumors, with the incidence peaking between the ages of 10 and 19. IGCTs are more common in males, with a male-to-female ratio of 4-5:1. These tumors typically occur in the pineal, suprasellar, or thalamic-basal ganglia regions, with lower frequencies in the third ventricle, brainstem, or corpus callosum. In Western countries, the incidence of iGCTs is 0.7 per million annually, compared to 2.7 per million annually in Asian countries (1, 2). According to the 2021 World Health Organization (WHO) classification of CNS tumors, iGCTs are classified into germinomas (GEs) and non-germinomatous germ cell tumors (NGGCTs). The latter category includes embryonal carcinoma, yolk sac tumor, choriocarcinoma, teratoma, and mixed germ cell tumors (3). GEs have a excellent prognosis, with a cure rate exceeding 90% (4). Whereas NGGCTs are more aggressive and associated with poorer outcomes, with a 5-year overall survival (OS) rate ranging from 50% to 90% (5–7). This stark difference in prognosis suggests underlying biological disparities that extend beyond histology.

Biologically, GEs are characterized by uniform primitive germ cells, genome-wide hypomethylation (8), and frequent mutations in the KIT/RAS/MAPK pathway (9, 10), reflecting a primordial germ cell origin. In contrast, NGGCTs are histologically heterogeneous, often showing hypermethylation and genetic alterations such as 12p gain (11–13) and PI3K/AKT pathway mutations (14–16), and HCG and AFP are often significantly elevated (17), indicative of dysregulated differentiation. In addition, the immune microenvironment is also different between the two subtypes, such as the expression of immune-related genes and the composition of immune cells and so on (18).

Clinically, iGCTs are treated using an multimodal therapy that includes radiotherapy, chemotherapy, and surgery. IGCTs are highly sensitive to both radiotherapy and chemotherapy, significant long-term toxicity remains a major concern. Radiotherapy can lead to late complications such as cognitive impairment, growth retardation, endocrine dysfunction, infertility, and secondary malignancies, particularly in the pediatric population (19–21). Similarly, chemotherapy is associated with late toxicities including endocrine, neurological, auditory, and psychiatric disorders (22, 23). Consequently, minimizing these treatment-related sequelae is crucial for preserving the long-term quality of life in survivors. These observations underscore the need for a deeper understanding of the underlying molecular abnormalities in these malignancies and the development of novel treatments with reduced toxicity, highlighting the urgency of advancing immunotherapy (5, 24).

Mounting evidence emphasizes the pivotal role of the tumor microenvironment, particularly the immune landscape, in modulating tumor progression and therapeutic response (25). A study has shown that higher degree of lymphocyte infiltration, (especially CD4+T cells, including helper T cells) showed better prognoses, while high expression of NOS2 (indicative of myeloid-derived suppressor cells or pro-tumor macrophages) was associated with worse prognoses in GEs (2), Similarly, independent studies have reported increased levels of cytotoxic T cells, activated cytotoxic T cells were significantly associated with favorable survival outcomes in iGCTs (26). Among immune cells, neutrophils have attracted increasing attention due to their prominent role in shaping the tumor microenvironment. Although their recruitment is known to play a central role in brain tumor pathogenesis, their specific function in iGCTs remains poorly explored (27). Neutrophils can undergo functional “reprogramming” within the tumor microenvironment, acquiring a pro-tumorigenic phenotype (28). Pro-tumor neutrophils promote tumor growth, angiogenesis, extracellular matrix remodeling, and immunosuppression. In addition, neutrophils actively participate in the activation of oncogenic signaling pathways, including STAT3, NF-κB, and Wnt/β-catenin, and enhance tumor stemness (29, 30). Stemness-associated programs are closely linked to invasive behavior, tumor recurrence, and poor clinical outcomes. A critical interface exists between neutrophils and cancer stemness, whereby neutrophil-derived inflammatory cytokines, and activate oncogenic signaling pathways, which are essential for maintaining cancer stem cell (CSC) self-renewal and survival (31, 32). This crosstalk suggests that neutrophils may be instrumental in sustaining the stemness, thereby contributing to tumor aggressiveness and progression.

In this study, we conducted a comprehensive study to investigate the interplay between cancer stemness and the immune microenvironment in iGCTs. First, we utilized transcriptome and methylation data of iGCTs to calculate stemness indices, and evaluated their association with clinical characteristics. Subsequently, we identified differentially expressed genes (DEGs) between GEs and NGGCTs, and the biological functions and signaling pathways involved in each subtype were delineated through functional enrichment analysis. Finally, we employed Gene Set Variation Analysis (GSVA) to quantify immune cell enrichment scores and performed correlation analysis to assess their relationship with the stemness indices. Our results demonstrated that the correlation between neutrophil infiltration and the stemness index was the most differentially significant feature between GEs and NGGCTs. Specifically, neutrophil counts were significantly higher in NGGCTs than in GEs.

To our knowledge, it is the first study to systematically integrate pre-chemotherapy peripheral blood neutrophil counts with multi-omics-derived stemness indices in iGCTs, establishing a framework that links systemic immunity, the tumor microenvironment, and molecular stemness to clinical outcomes. Consequently, tumor-associated neutrophils emerge not only as a crucial prognostic factor but also as a promising target for novel therapeutic strategies.

## 2 Materials and methods

### 2.1 Patients and samples

Peripheral blood samples used in this study were obtained from iGCT patients at Tiantan Hospital between January 2019 and December 2022, including 58 GE and 33 NGGCT patients (detailed information about these samples in **Supplementary Table 1**). The peripheral blood samples were taken from the iGCT patients prior to chemotherapy. The diagnosis was confirmed by pathology. None of these patients had acute conditions such as bacterial or viral infections, or drug treatments that might affect the immune system, as per the hospital case records. This study was approved by the institutional review board of Beijing Tiantan Hospital, and written informed consent was obtained from all patients.

### 2.2 Public clinical and molecular data collection

Gene expression microarray data, 450K methylation data, and copy number variation (CNV) data of iGCT cohorts, along with corresponding clinical information, were downloaded from Gene Expression Omnibus (GEO) (8, 33). Additionally, gene expression microarray data for chorionic villus samples, leaf chorionic samples from postpartum placental tissue, and their clinical information were also downloaded from the GEO database.

A total of 1113 immuno-related human genes, including 24 immune cells, and 27 immune response categories (which include angiogenesis and tumor invasion), that were curated from the nCounter^®^ PanCancer Immune Profiling Panel (NanoString) were used as candidate genes in this study. Detailed annotations for these immune cell and immune response-related genes are provided in **Supplementary Tables 2**, **3**.

### 2.3 Calculating mRNAsi and mDNAsi

We calculated the mRNA Expression-Based stemness index(mRNAsi) and DNA Methylation-Based stemness index(mDNAsi) using the “one-class logistic regression (OCLR)” algorithm (34). The mRNAsi was calculated using RNA-seq data from 13 iGCT patients. The mDNAsi was derived from methylation array profiles of 69 iGCT patients. The mRNAsi and mDNAsi value range from 0 to 1, with a higher stemness capacity indicated by values closer to 1. To evaluate the robustness of the stemness indices, Principal Component Analysis (PCA) was conducted on the transcriptomic and methylation data of iGCT and placental villus samples, clustering them to observe correlations and disparities. The impact of mRNAsi and mDNAsi on patients outcome was assessed using Kaplan-Meier survival analysis for OS. Differences in survival curves between groups were compared using the log-rank test.

### 2.4 Identification and functional enrichment analysis of DEGs

To investigate the biological processes in GEs and NGGCTs, we first used the “limma” R package to identify DEGs between the two groups. Based on Bayesian calculation of T-values, F-values and log-odds, the eligible DEGs were selected using the criteria of |log2(FC)|> 1.5 and adj P Value < 0.05. DEGs were visualized using volcano plots generated with the ggplot2 package in R. We then used the “clusterProfiler” R package to perform Gene Ontology (GO) and Kyoto Encyclopedia of Genes and Genomes (KEGG) an clusterProfiler alyses.

### 2.5 Regulation of DEGs in the iGCTs

We identified DEGs in iGCTs and explored the mechanisms underlying changes in gene expression through epigenetic variations and CNVs. To study the relationship between gene expression and DNA methylation, we mapped DNA methylation probes to the corresponding genes. The methylation level of a gene was defined as the mean value of all probes mapping to it. The beta value for each gene was evaluated within each immune subtype. We also used Student’s t-test to examine whether these genes were differentially methylated in GEs compared to NGGCTs. For CNVs, deletion is represented by negative values, with smaller values indicating greater reductions in copy number. Conversely, amplification was represented by positive values, with larger values indicating greater increases in copy number. We used Student’s t-test to examine whether these genes had copy number changes in GEs compared to NGGCTs.

### 2.6 Immune cell subpopulations and responses in iGCTs

We used gene set enrichment analysis to identify immune cell types in GEs and NGGCTs. The expression level of each gene was log2‐transformed for subsequent analysis. For each patient, genes were ranked in descending order based on their expression values, and the association was represented by a normalized enrichment score. An immune cell type was considered enriched in a patient if the P‐value was < 0.1.

The enrichment scores of immune responses in each immune subtype were determined by single sample enrichment analysis in the R package “GSVA”. GSVA is used to estimate the variation in gene set enrichment through samples of expression datasets, so the enrichment scores of immune responses in each subtype to be computed can be compared (35).

Nest, we performed Pearson correlation analysis to evaluate the relationship between mRNAsi and the immune microenvironment. The Pearson correlation coefficient quantifies the strength and direction of the relationship, ranging from -1 to 1. Positive values indicate a positive correlation, while negative values indicate a negative one. The closer the value is to 1 or -1, the stronger the correlation.

### 2.7 Peripheral blood immune cells in iGCT patients

We used the Student’s t-test to assess the count and proportion of leukocytes and five leukocyte subtypes (neutrophils, lymphocytes, monocytes, eosinophils, and basophils) in the peripheral blood of iGCT patients. Results were visualized using the ggplot2 package in R.

### 2.8 Statistical analysis

All statistical analyses were performed using R software (http://www.r-project.org). Heatmaps and Circos plots were generated using the R packages pheatmap and OmicCircos, respectively. All statistical tests were two-sided, with a p-value of less than 0.05 considered statistically significant unless otherwise specified.

## 3 Results

### 3.1 The stemness index in iGCT patients

**Figure 1:**
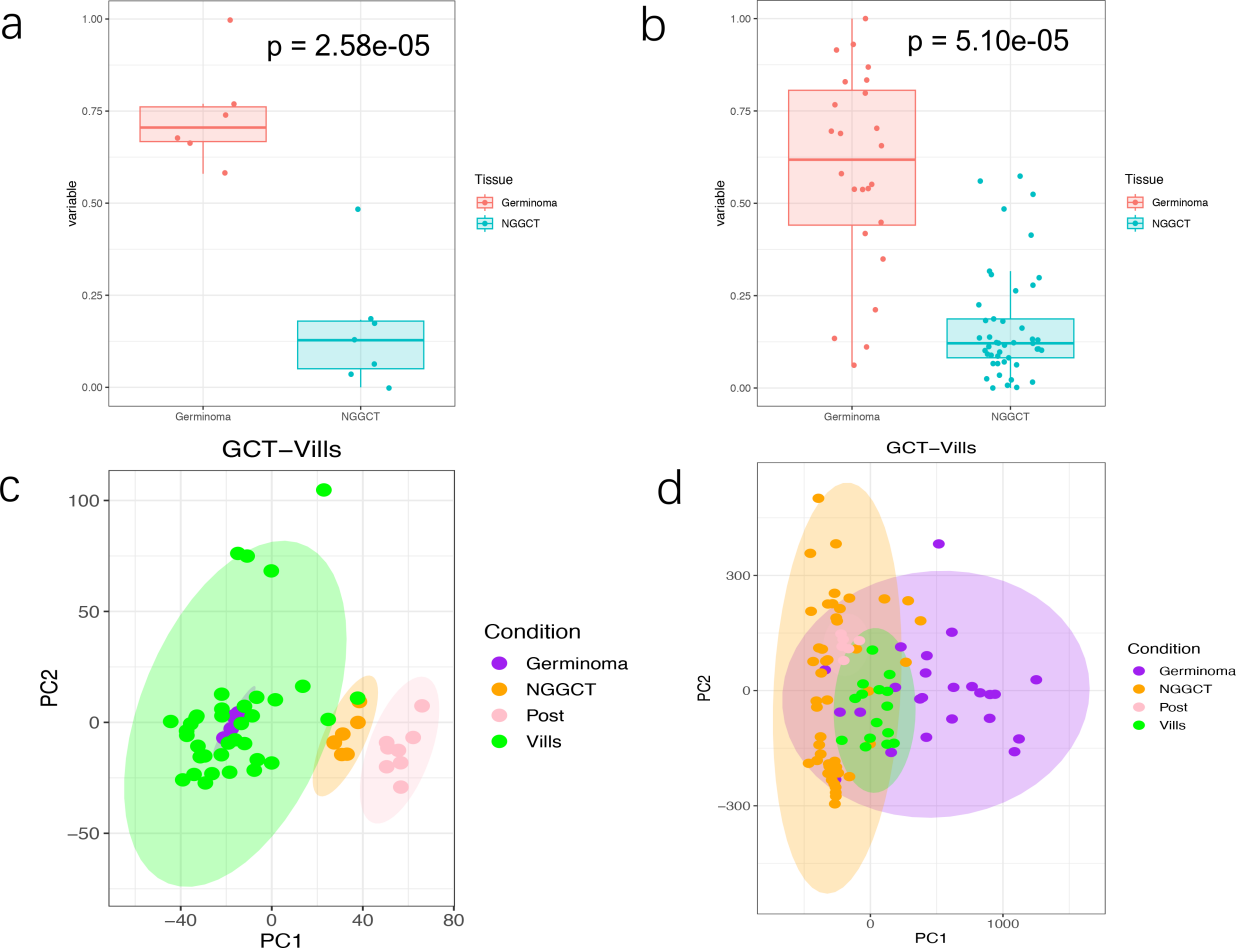
The stemness index in GE and NGGCT patients. **(A, B)** Boxplots showing the mRNAsi and mDNAsi across the two subtype, respectively. The central line represents the median, the box edges represent the interquartile range (IQR), and the whiskers extend to 1.5×IQR. Dots indicate outliers. Statistical significance was assessed using Student’s t-test. **(C, D)** PCA plot based on the normalized gene expression profiles and β-values of methylation profiles of four sample groups: GE samples (purple), NGGCT samples (orange), postpartum placental samples (pink) and villi samples (green). Each point represents an individual sample, with ellipses showing the 95% confidence intervals for each group. The clustering suggests distinct transcriptional profiles across groups.

Using the OCLR algorithm, we found that mRNAsi was significantly higher in GE tissues than in NGGCT tissues (p = 2.58e-05, **Figure 1A**), and the mDNAsi exhibited a similar trend (p = 5.10e-08, **Figure 1B**). PCA on iGCTs and chorionic samples (previously published (36)), revealed that GEs clustered with chorionic villus samples, while NGGCTs clustered with postpartum placental samples, both in expression profiles (**Figure 1C**) and methylation data (**Figure 1D**). This suggests that GEs are closely related to cells at an early stage of germ cell development, while NGGCTs are linked to more differentiated cells, consistent with previous findings (37).

As is well known, GE patients have a better prognosis than NGGCT patients. Our survival analysis of both transcriptomic and methylation data revealed a congruent pattern. The methylation data was significantly associated with poorer prognosis in NGGCTs (p= 0.046, **Supplementary Figure 1B**), while the transcriptome data showed a non-significant trend in the same direction (**Supplementary Figure 1A**). Next, The Kaplan-Meier analysis showed that a lower mDNAsi was significantly associated with worse OS (p= 0.037, **Supplementary Figure 1D**), whereas patients in the mRNAsi-low group showed a similar, though not significant, trend toward poorer outcomes (**Supplementary Figure 1C**). The transcriptome data and the mRNAsi were not significantly associated with OS in iGCT patients, which may be related to the small cohort size and should be explored in future research.

### 3.2 Correlation between stemness index and clinicopathological features of iGCT patients

**Figure 2:**
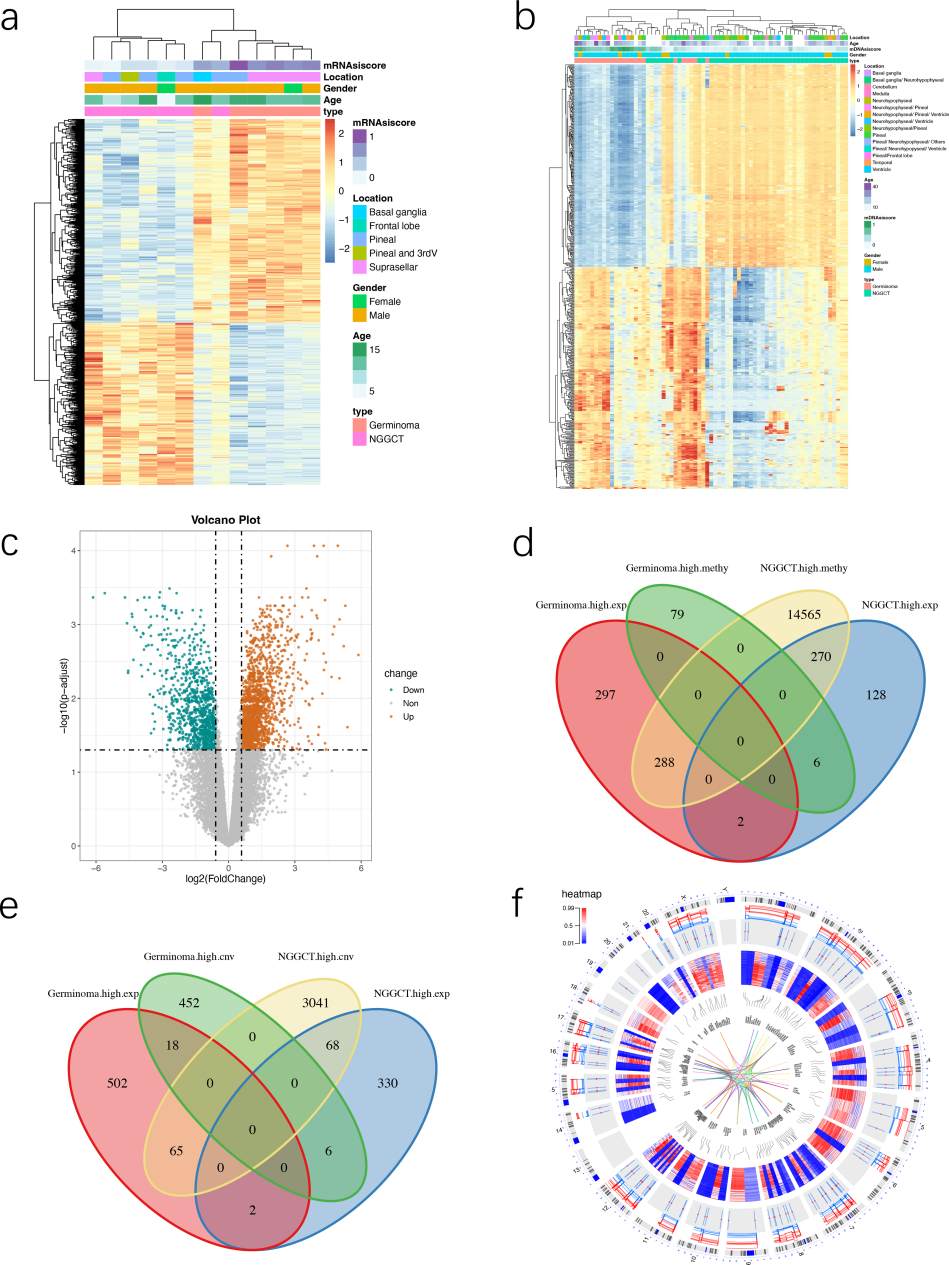
Identification of DEGs and regulation between multiple omics. **(A, B)** Heatmap showing mRNAsi and mDNAsi across different clinical characteristics, including sex, age and tumor locations. Rows represent gene expression, methylation expression, respectively; and columns represent samples. Colors in the heatmap correspond to different levels of expression in the samples, with red indicating high expression and blue indicating low expression. The hierarchical clustering highlights patterns of expression associated with these clinical variables. **(C)** Volcano plot visualized the DEGs between GE and NGGCT samples. The salmon and cyan points represent the DEGs with statistical significance, salmon represents upregulated genes in GEs, and cyan represents upregulated genes in NGGCTs. **(D)** Venn diagram showing the intersection of aberrantly expressed genes and methylated genes. **(E)** Venn diagram showing the intersection of aberrantly expressed genes and CNV genes. **(F)** Circos plot displaying the distribution of aberrant genes in transcriptome, CNV, methylation data, and their interactions on chromosomes.

We obtained 13 mRNAsi samples and 69 mDNAsi samples, both of which showed a clear association with survival. We then explored the relationship between mRNAsi and mDNAsi with clinical characteristics of patients. The heatmap displays the gene expression and methylation profiles across the samples. Each row represents gene characteristics, and each column represents samples. These annotations column categorize each sample by: type (GEs *vs*. NGGCTs), age, sex, tumor location, and stemness scores (**Figure 2A**-2B). This integrated visualization allows for the simultaneous assessment of gene clusters and their potential associations with key sample characteristics. Through correlation analysis, we obtained that no significant difference between gender and mRNAsi and mDNAsi. Additionally, tumor location showed no notable relationship with mRNAsi or mDNAsi. However, age showed a positive correlation with both mRNAsi (**Supplementary Figures 2A**, R = 0.52, p= 0.07) and mDNAsi (**Supplementary Figures 2B**, R = 0.38, p= 0.0037).

### 3.3 Identification of DEGs and functional enrichment analysis

We identified 900 DEGs, including 501 genes highly expressed in GE patients and 399 genes highly expressed in NGGCT patients (**Figure 2C**). Additionally, 15,208 differentially methylated genes were identified, with 85 showing higher methylation levels in GE patients and 15,123 showing higher methylation levels in NGGCT patients. Through comparison, we found that there are 288 common genes in GEs that are highly expressed and have low methylation levels, whereas there are 6 common genes in NGGCTs that are highly expressed and have low methylation levels (**Figure 2D**). For CNV data, we identified 365 genes with CNVs, of which 65 showed increased copy numbers in GE patients and 3,174 showed increased copy numbers in NGGCT patients. In GEs, 18 genes had both high expression and increased copy numbers, while NGGCTs had 68 genes with both high expression and increased copy numbers (**Figure 2E**). The changes in the transcriptome expression, methylation and CNV levels of these genes are shown on chromosomes. In the circos plot, the ideogram of a normal karyotype is shown in the outermost ring. The next ring shows the gene expression heatmap at corresponding genomic coordinates, with red indicating high expression and blue indicating low expression. The following ring represents CNVs, with stair-step lines showing different individuals; red lines indicate copy number amplification and blue lines indicate copy number deletion. The innermost ring shows the DNA methylation β values heatmap, with red representing high methylation and blue representing low methylation. The middle ring shows gene-gene interactions (**Figure 2F**). These findings suggest that changes in copy number and methylation may influence gene expression levels. However, the regulatory complexity varies between different genes, requiring further exploration.

**Figure 3:**
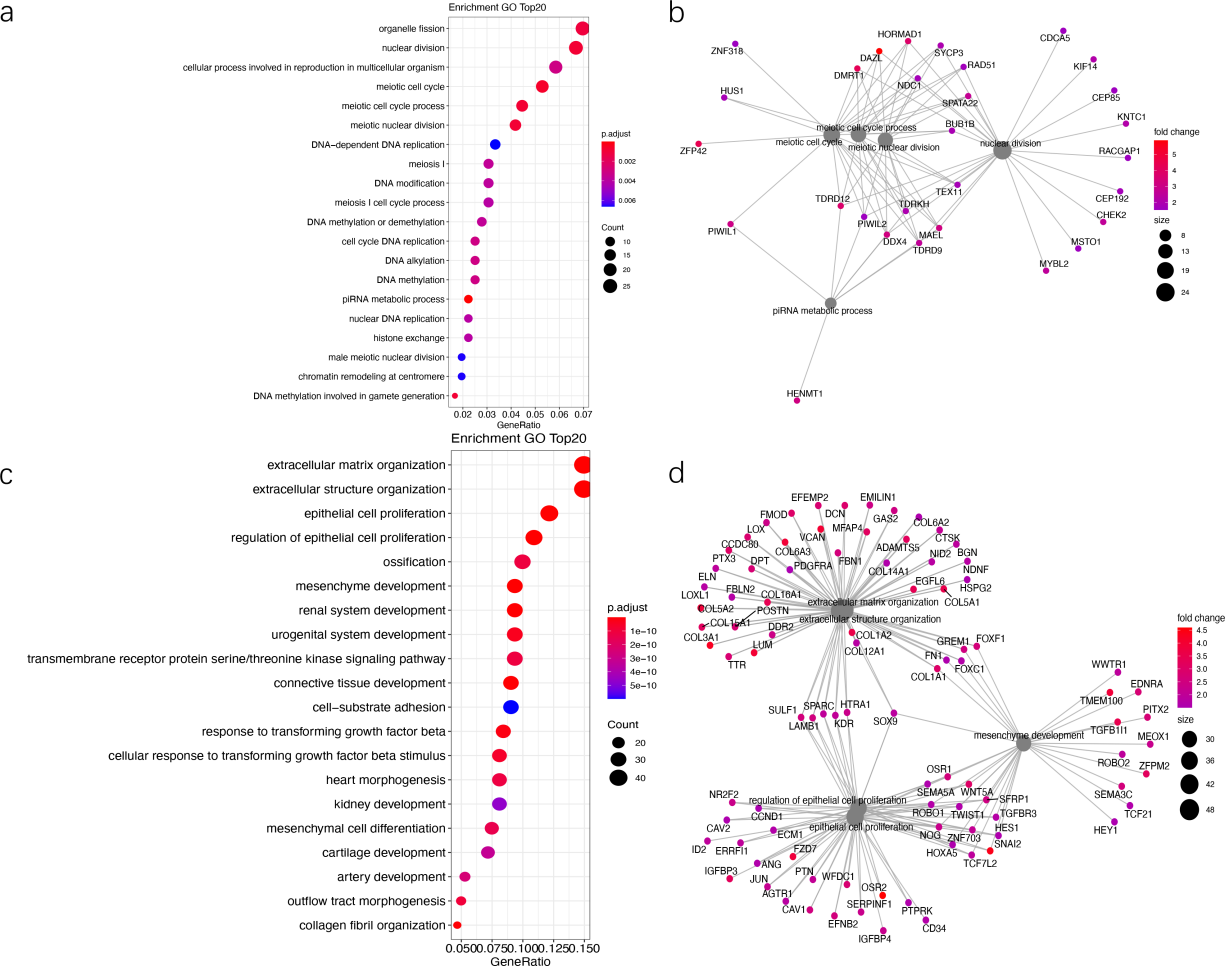
Functional enrichment analysis of DEGs. **(A, C)** Bubble plots showing the top 20 GO terms in biological process analyses for upregulated genes in GEs and NGGCTs, respectively. GO terms are ranked based on the -log10 (p-value), indicating statistical significance of enrichment. The size of each bubble represents the number of genes enriched in the respective GO term, while the color gradient reflects the adjusted p-value or enrichment score, with red indicates higher significance, and blue indicates lower significance. **(B, D)** KEGG pathway maps visualizing significantly altered pathways for upregulated genes in GEs and NGGCTs, respectively. Nodes represent genes involved in the pathway, with colors indicating their differential expression. The size of the nodes reflects the significance of the pathways, while edges represent molecular interactions or regulatory relationships.

Next, the results of functional enrichment analysis showed that the genes highly expressed in GEs are mainly involved in cell proliferation-related processes, such as organelle fission, nuclear division, and DNA-dependent DNA replication. The top 20 significantly enriched processes are shown in **Figure 3A**. The pathways involving these highly expressed genes include meiotic cell cycle processes, nuclear division, and meiotic nuclear division, as shown in **Figure 3B**. Genes highly expressed in NGGCTs are primarily enriched in functions related to extracellular matrix organization, mesenchyme development, and extracellular structure organization. The top 20 most significantly enriched functions are shown in **Figure 3C**. These highly expressed genes are also involved in pathways such as mesenchyme development, regulation of epithelial cell proliferation, and epithelial cell proliferation, as shown in **Figure 3D**. Although the stemness of GEs is higher than NGGCTs, NGGCTs are more malignant and have a poorer prognosis, indicating the involvement of other factors in tumor progression. This suggests that stroma-related biological behaviors within the tumor microenvironment may facilitate NGGCT initiation and progression.

### 3.4 Correlation between mRNAsi and immune cells

**Figure 4:**
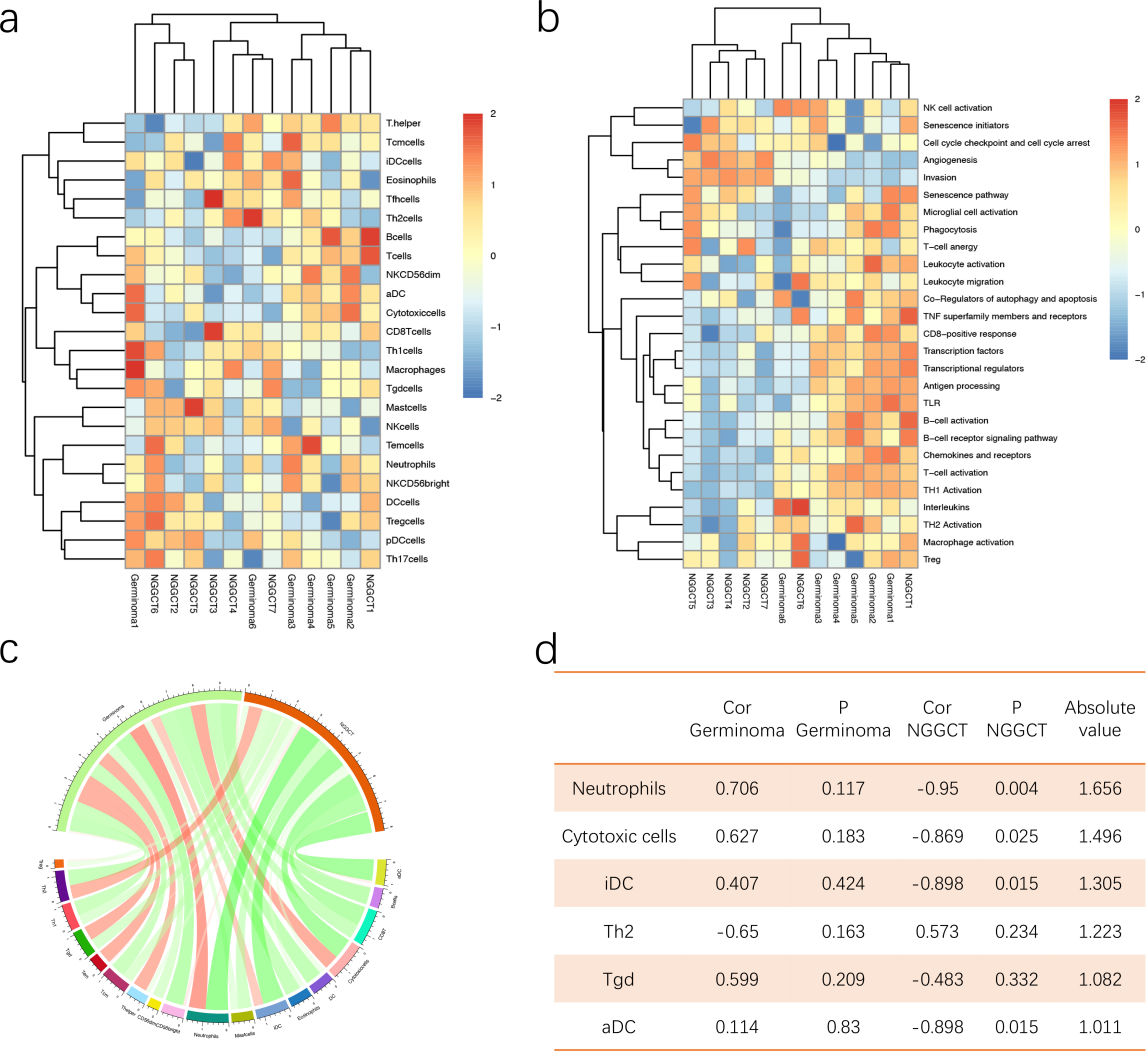
Regulation of mRNAsi and the tumor microenvironment. **(A)** Heatmap of GSVA scores for immune cell enrichment in GE and NGGCT patients. Rows represent different immune cell types, and columns represent individual samples grouped into GE and NGGCT categories. Color intensity reflects the relative enrichment score of immune cells, with red indicating high enrichment and blue indicating low enrichment. Hierarchical clustering was performed on both rows and columns to identify immune cell infiltration patterns. **(B)** Heatmap of GSVA scores for immune responses in GE and NGGCT patients. **(C)** Chord diagram illustrating the relationships between mRNAsi and immune cell enrichment scores. Each segment in the outer circle represents an immune cell type. The width of the chords connecting the segments reflects the strength of the association between stemness indices and immune cell scores, as measured by correlation coefficients. Positive correlations are shown in red, while negative correlations are displayed in green. **(D)** The correlation coefficients and statistical values of mRNAsi associated with representative immune cells in GEs and NGGCTs are shown in the table.

To evaluate whether tumor microenvironment features influence outcomes, we analyzed the microenvironments across all iGCT samples. The results showed that immune cell subpopulations differed between GEs and NGGCTs. In GEs, the top three immune cell subpopulations with the highest enrichments included cytotoxic cells, NKCD56dim, and aDC. In NGGCTs, the top three highest enrichments included mast cells, Treg cells, and DC cells. The scores of these immune cell subsets and the corresponding statistical differences between the two groups are summarized in **Supplementary Table 4**, and these findings are illustrated in **Figure 4A**. These results indicated the immune cells in the tumor immune microenvironment of different subtypes are different, suggesting that the microenvironment affects the prognosis of patients.

Besides the differences in immune cell subpopulations, GSVA revealed distinct immune responses between GEs and NGGCTs. GEs showed high enrichment scores in T-cell activation, CD8-positive response and Antigen processing, while the NGGCTs exhibited high enrichment scores in Invasion, Angiogenesis, and other processes. The enrichment scores for these immune responses and their corresponding statistical differences are presented in **Supplementary Table 5**, collectively illustrated in **Figure 4B**.

The correlation between mRNAsi and the enrichment scores of 24 immune cells is shown in **Figure 4C**. Among them, the correlation between mRNAsi and neutrophils enrichment score in GEs is 0.706 (p= 0.117), while in NGGCTs, it is -0.95 (p= 0.004), indicating a strongly positive and strongly negative correlation, respectively. The most significant correlation difference was observed for neutrophils (**Figure 4D**). These results indicated that neutrophils in the tumor microenvironment play a crucial role in iGCT development and progression.

### 3.5 Peripheral blood immune cells in iGCT patients

We collected peripheral blood samples from 58 GE and 33 NGGCT patients, including 21 females and 70 males, aged 5 to 32 years, with a median age of 13. Pineal and sellar lesions were the most common locations (**Table 1**).

**Table 1:** Summary of GCT patients information.

Clinicopathologic	Number	Percent (%)
Gender Male Female	70 21	76.9 23.1
Age ≤ 10 10 - 15 > 15	24 35 32	26.4 38.5 35.1
Pathological type Germinoma NGGCT	58 33	63.7 36.3
Location Pineal Sellar Basal ganglia Pineal & Sellar Thalamus Other	37 25 7 4 3 15	40.6 27.5 7.7 4.4 3.3 16.5

**Figure 5:**
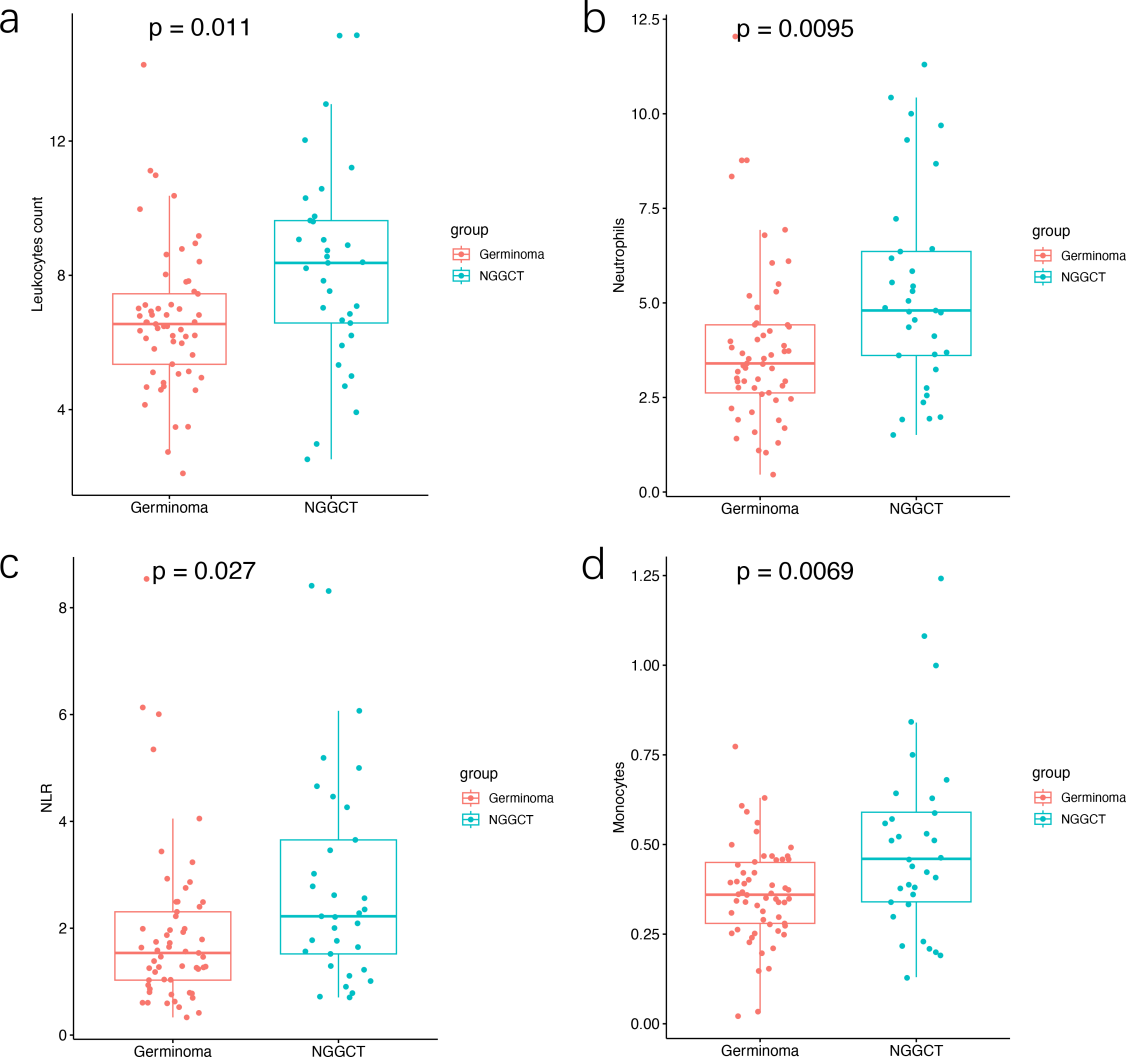
Boxplot comparing preoperative peripheral blood content among iGCT patients. **(A)** Leukocyte count differed between GE and NGGCT samples (p= 0.011). **(B, C, D)** Neutrophil counts, NLR, and monocyte proportions differed between GEs and NGGCTs (p= 0.0095, p= 0.027, p= 0.0069), respectively. Pairwise comparisons between groups were performed using the unpaired Student’s t-test.

We assessed the leukocyte count, as well as the count and proportion of five leukocyte subtypes (neutrophils, lymphocytes, monocytes, eosinophils, and basophils) in the peripheral blood of iGCT patients. As shown in **Figure 5**, NGGCTs had significantly higher leukocyte counts compared to GEs (p= 0.011). Neutrophils showed a similar trend. Neutrophil counts in NGGCTs were significantly higher than in GEs (p= 0.0095). The NLR (Neutrophil‐to‐lymphocyte ratio) in NGGCTs was significantly higher than in GEs (p= 0.027). Monocyte counts were significantly higher in NGGCTs than in GE patients (p= 0.0069). However, no statistically significant difference was observed for lymphocyte, eosinophil, or basophil counts (**Supplementary Figures 3A–C**).

## 4 Discussion

We conducted an in-depth analysis of the relationship between the stemness of iGCTs and immune response, identifying neutrophils as being associated with stemness and playing a key role in iGCTs. This was verified using patient samples and clinical data from our cohorts.

Increasing evidence suggests that stemness and the immune microenvironment have emerged as important features of cancer, with their interactions affecting tumor development and prognosis (38). Research on immune-related characteristics has gained significant attention. However, the relationship between stemness and immune microenvironment has not been extensively explored in iGCTs. Further research on how to guide clinical treatment and predict patient prognosis holds considerable potential.

In this study, using RNA-seq, and methylation datasets of iGCTs, survival analysis showed that GEs had a better prognosis than NGGCTs, consistent with previous reports (39, 40). By employing the OCLR algorithm, we revealed a significantly higher stemness index in GEs than NGGCTs. This may be related to the “embryonic cell theory” of iGCT pathogenesis, which suggests that mis-migrated pluripotent embryonic cells, at an early developmental stage prior to primordial germ cell progression, may give rise to iGCTs (8, 41).

The stemness index was significantly associated with patient prognosis and positively correlated with age. However, it was not significantly related to gender or tumor location. Due to the small sample size, further analysis with larger cohorts or external datasets is needed. To further investigate the differences between GEs and NGGCTs, we identified DEGs, as well as variations in CNV and methylation between the two groups. In GEs, genes such as *RASL10A*, *ABCC2*, *SLC4A11*, *E2F6*, *LOC100130331*, *IER5*, *RBM46*, *BUB1B*, *ZNF239*, *ELOVL3*, *CTSS*, and *PPP1R14C* were highly expressed, hypomethylated, and showed increased copy numbers. Many of these genes play critical roles in tumorigenesis. In contrast, no genes in NGGCTs were identified with high expression, low methylation, and increased copy numbers. These findings suggest that changes in CNV and methylation impact gene expression levels. Further work is needed to explore the functional implications of these associations.

GEs exhibit higher stemness indices, and they were characterized by an immune contexture enriched in T-cell activation, CD8-positive response and antigen processing, consistent with previous reports (42), which are generally associated with effective antitumor immune responses and favorable prognosis. This immune-active microenvironment may counterbalance intrinsic stemness programs, thereby limiting malignant progression and contributing to the excellent treatment responsiveness observed in germinomas. In contrast, NGGCTs demonstrate relatively lower stemness, but were enriched in invasion, angiogenesis and extracellular matrix remodeling, accompanied by increased neutrophil infiltration, aligning with prior studies (43, 44). This immunosuppressive microenvironment promotes the occurrence and development of tumors.

Notably, we observed a stronger differences in the interactions of immune cells and stemness indices in GEs and NGGCTs. In GEs, the interaction coefficient between stemness indices and immune cells was higher than in NGGCTs, disordered interactions between immune cells and stemness indices affect the immune microenvironment, thus impacting patient prognosis. Based on immune cell-stemness interactions, we identified neutrophils as the immune cells with the greatest correlation differences between GEs and NGGCTs, suggesting that neutrophils may contribute to the maintenance or enhancement of aggressive tumor phenotypes in NGGCTs. Accumulating evidence suggests that neutrophils can transform into tumor-promoting phenotype and promote the occurrence and development of tumors (29, 45). Neutrophils have been shown to promote tumor aggressiveness by activating oncogenic signaling pathways, as well as releasing reactive oxygen species, neutrophil extracellular traps, and proinflammatory cytokines. These neutrophil-mediated signals are essential for maintaining CSC self-renewal, plasticity, and resistance to radiotherapy and chemotherapy (31, 32, 46). These findings suggest that the clinical outcomes of iGCTs are influenced not only by tumor stemness but also by subtype-specific immune–stemness interactions, with neutrophils may represent a key modulatory component linking the immune microenvironment to malignant potential, particularly in NGGCTs.

Furthermore, we evaluated peripheral blood leukocyte counts in iGCT patients using our data. Neutrophil counts were significantly higher in NGGCTs than in GEs, and neutrophils are considered a response to systemic inflammation. Neutrophil and lymphocyte recruitment plays a critical role in brain tumors (27). Additionally, the NLR is a prognostic marker for various solid tumors (47, 48), requiring validation with comprehensive survival data. To further elucidate the mechanistic role of neutrophils in iGCTs, future studies will include the isolation of neutrophils from peripheral blood samples for transcriptomic profiling and T-cell receptor (TCR) sequencing analyses.

Our study has some limitations. First, the histological samples were small and not sufficiently representative, and a prospective study with a larger cohort is needed. Second, our analyses were limited to data from molecular assays, lacking classical cellular immunology assays to confirm cell phenotype distribution. In addition, peripheral blood cell counts are easily influenced by factors such as chronic diseases, localized or systemic infections, and any medications associated with the patient’s inflammatory status, and the follow-up time for our patients is short, and the endpoint has not yet been reached.

In summary, we present an integrative analysis of tumor stemness and immune microenvironment heterogeneity in iGCTs. Using multi-omics data, we demonstrate that GEs and NGGCTs display distinct stemness and immune landscapes, which may contribute to their markedly different clinical outcomes. Importantly, we identify a previously underexplored association between neutrophils and cancer stemness, suggesting a potential role for neutrophils in modulating tumor aggressiveness. Our findings further indicate that peripheral blood neutrophil counts may serve as a readily accessible prognostic biomarker, providing a template for investigations of iGCTs as well as other rare malignancies, and warrant further experimental validation and prospective clinical studies.

## Data Availability

The original contributions presented in the study are included in the article/**Supplementary Material**. Further inquiries can be directed to the corresponding author.

## Supplementary material

Supplementary Figure 1 Kaplan-Meier survival curves comparing survival between GE and NGGCT patients. (A-B) Kaplan-Meier curves of transcriptome data and methylation Data in iGCT patients, respectively. The cyan line represents the GE group, whereas the salmon line represents the NGGCT group. (C-D) Kaplan-Meier curves of mRNAsi and mDNAsi scores in iGCT patients, respectively. The cyan line represents the low-stemness index group, while the salmon line represents the high-stemness index group. Statistical significance was assessed using the log-rank test.

Supplementary Figure 2 Scatter plot showing the correlation between age and stemness index. (A) The relationship between age and mRNAi shows a positive correlation, but it is not statistically significant (R = 0.52, p= 0.07). (B) The relationship between age and mDNAsi shows a significant positive correlation (R = 0.38, p= 0.0037). The solid regression line highlights the relationship between variables, with the shaded area indicating the 95% confidence interval. The Pearson correlation coefficient and p-value are displayed on the plot.

Supplementary Figure 3 Boxplot comparing preoperative peripheral blood content in iGCT patients. (A, B, and C) Comparison of lymphocytes, eosinophils, and basophils between GE and NGGCT Samples, but no significant statistical differences were observed (p> 0.05).

Supplementary Table 1 The detailed information on our GE and NGGCT patients.

Supplementary Table 2 Gene signatures for immune cell types.

Supplementary Table 3 Gene signatures for immune response categories.

Supplementary Table 4 Statistical comparison of immune cell types enrichment scores between GE and NGGCT samples.

Supplementary Table 5 Statistical comparison of immune response categories enrichment scores between GE and NGGCT samples.
