# Supplementary figures and images for: Crosstalk of cancer stemness-neutrophils in outcome of intracranial germ cell tumors
Source: Front Immunol. 2026 Mar 5;17:1571513. doi: 10.3389/fimmu.2026.1571513 (PMC12999580; doi:10.3389/fimmu.2026.1571513)

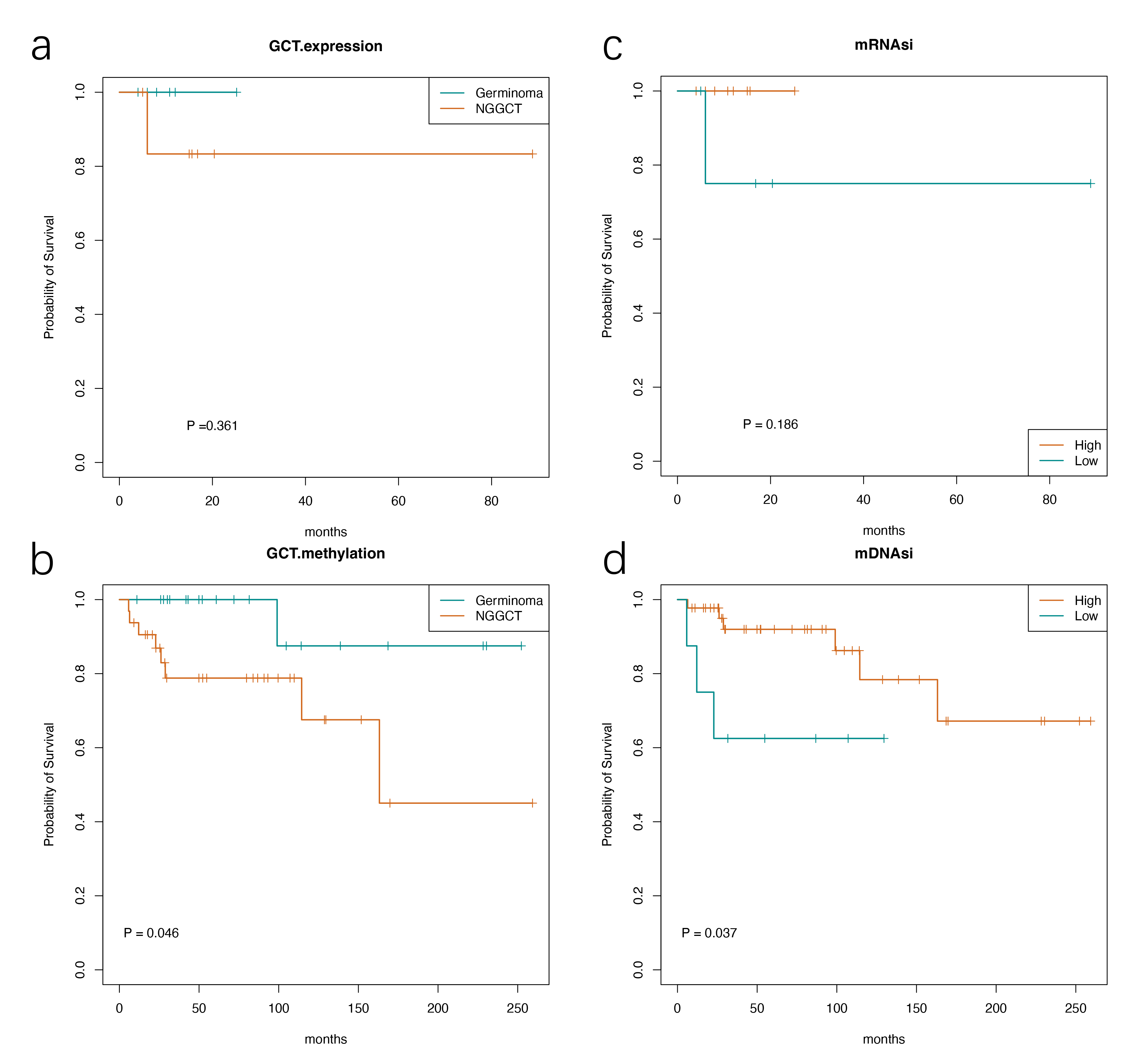

Supplement: Supplementary Figure 1 — Kaplan-Meier survival curves comparing survival between GE and NGGCT patients. (A-B) Kaplan-Meier curves of transcriptome data and methylation Data in iGCT patients, respectively. The cyan line represents the GE group, whereas the salmon line represents the NGGCT group. (C-D) Kaplan-Meier curves of mRNAsi and mDNAsi scores in iGCT patients, respectively. The cyan line represents the low-stemness index group, while the salmon line represents the high-stemness index group. Statistical significance was assessed using the log-rank test. [file Image1.tif]

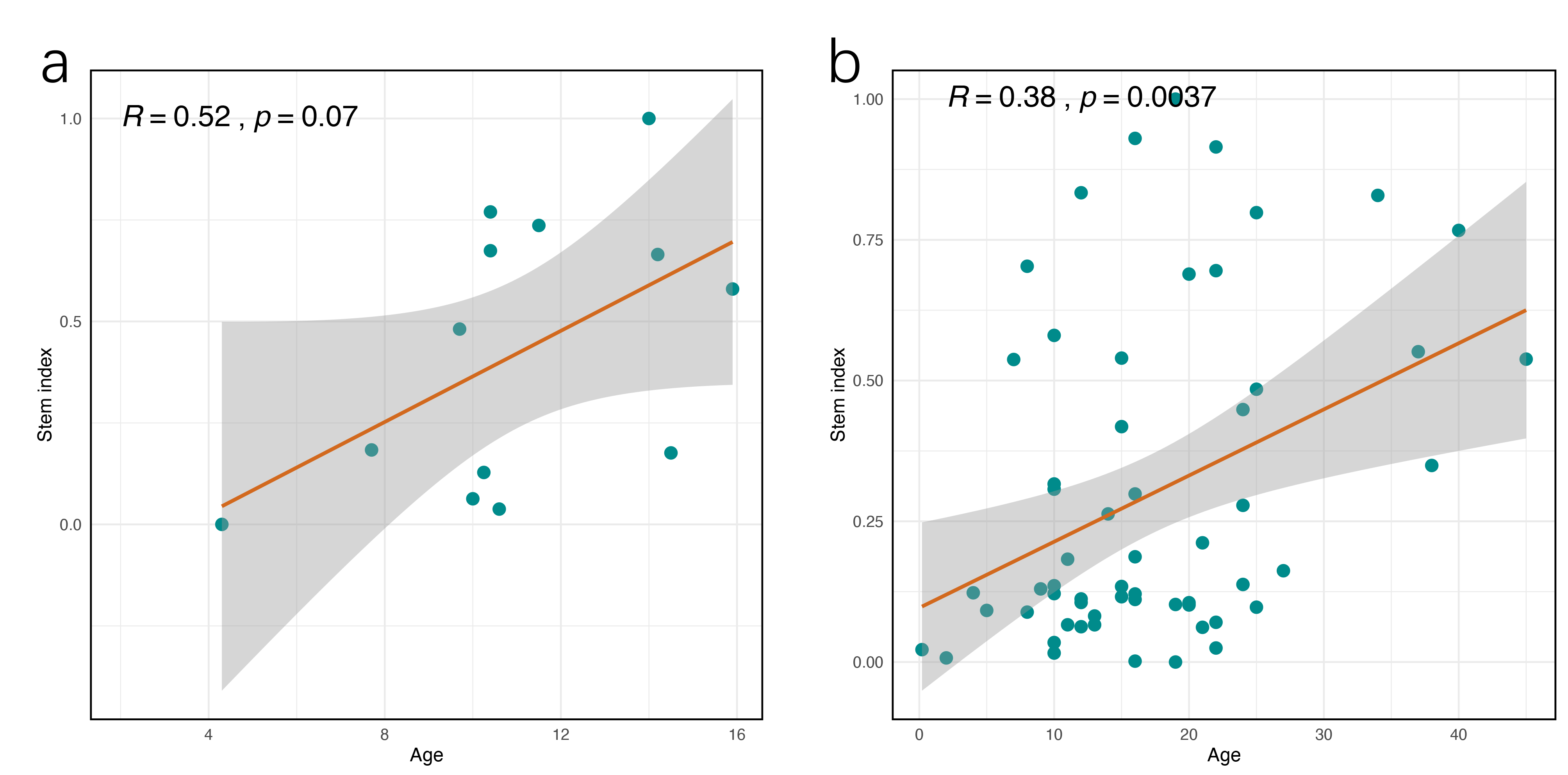

Supplement: Supplementary Figure 2 — Scatter plot showing the correlation between age and stemness index. (A) The relationship between age and mRNAi shows a positive correlation, but it is not statistically significant (R = 0.52, p= 0.07). (B) The relationship between age and mDNAsi shows a significant positive correlation (R = 0.38, p= 0.0037). The solid regression line highlights the relationship between variables, with the shaded area indicating the 95% confidence interval. The Pearson correlation coefficient and p-value are displayed on the plot. [file Image2.tif]

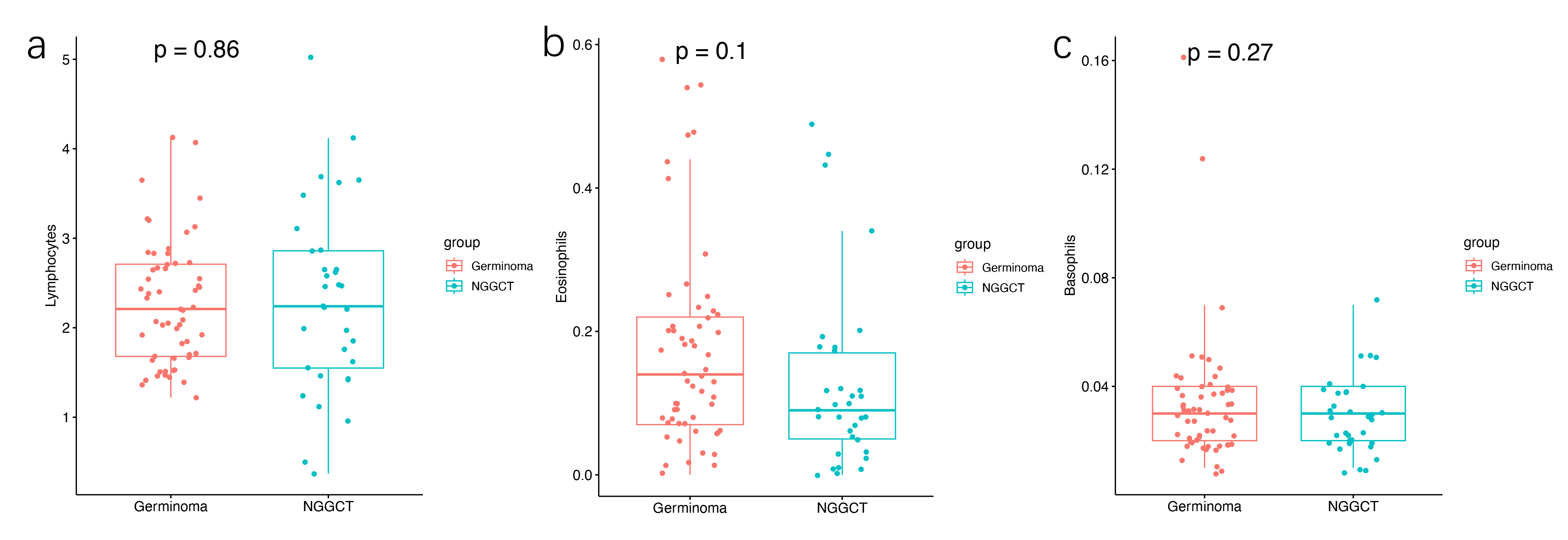

Supplement: Supplementary Figure 3 — Boxplot comparing preoperative peripheral blood content in iGCT patients. (A, B, and C) Comparison of lymphocytes, eosinophils, and basophils between GE and NGGCT Samples, but no significant statistical differences were observed (p> 0.05). [file Image3.tif]
